# Behind the scenes at *The Plant Cell*: The process from prereview consultation to an editorial decision

**DOI:** 10.1093/plcell/koac233

**Published:** 2022-07-29

**Authors:** Blake C Meyers

**Affiliations:** Editor-in-Chief, *The Plant Cell*, American Society of Plant Biologists, USA; Donald Danforth Plant Science Center, St. Louis, Missouri, USA; Division of Plant Science and Technology, University of Missouri-Columbia, Columbia, Missouri, USA

Authors may wonder how manuscripts are considered and evaluated at *The Plant Cell*. A particular “black box” may be the prereview consultation session, as this important stage in the evaluation of their submissions is obscured by the authors and reviewers. What is the process of manuscript consideration, and how do we determine whether a manuscript is sent for external review? This process builds on the work of my predecessor, former Editor-in-Chief Sabeeha Merchant (see Merchant, 2015) who implemented it at *The Plant Cell*. Prior to her tenure, roughly 90% of manuscripts were sent to external review—a level that risked reviewer fatigue. Since implementing the prereview consultation process in 2015, we now decline about 50% of submissions without external review. These manuscripts are assessed (to some extent “reviewed”) by multiple editors, and the letter explaining the decline decision aims to provide specific details about the assessment, reducing the number of rounds of review for many manuscripts. We also aim to make the decision about external review quickly; the median turnaround from submission to decline without external review over the last 18 months was 7 days. For these manuscripts declined without external review, the letter should clearly state whether or not we will reconsider the work and the extent of revisions that would be needed. We often encourage authors to submit elsewhere for more rapid publication.

Manuscripts that are submitted to *The Plant Cell* first go through an internal check to ensure the files are in order. Until recently, this was handled by our excellent but now retired Manuscript Manager, Annette Kessler (Goldberg et al., 2022). Once all authors have approved the submission and it passes this quality control step, the manuscript lands in my queue, as Editor-in-Chief, for assignment to a Senior Editor. In my role, I review the author list to check for possible conflicts of interest by board members, including institutional conflicts. I read the cover letter to understand the nature of the work and its impact, and to understand any unusual circumstances associated with the submission, such as competing or coordinated papers, or recent, related publications. The cover letter is an important component of the submission—it is a first chance to make the case for your work in plain language. Although I’m a plant biologist, my expertise is perhaps no broader than average and many manuscripts are out of my area, but I do read many cover letters and papers. Can you make your work stand out, even if I don’t know your topic well? If a compelling story, notable point, or otherwise unusual aspect of the letter catches my eye, I will relay this to the Senior Editor. They will identify the stand-out papers too, as they also read the cover letter, and since they’ll have expertise more directly associated with the work, they may have a keener appreciation of topical points. Recognition from more individuals noticing your work because of a clear cover letter may help move the paper through this stage in the process. I also read over the manuscript. Since we receive over 900 new submissions per year, I focus mainly on the figures unless the paper is directly in my field. As the old adage says, a picture is worth a thousand words. Can I quickly get the gist of the paper from the figures, and does this match the enthusiasm I take from the cover letter? Or are the figures disappointing, poorly conceived and executed, or difficult to interpret? These are messages I might convey as I select one of our experienced Senior Editors to handle the manuscript.

When a Senior Editor accepts an assignment, their first step is like mine—read the cover letter and manuscript, and then decide how to proceed. If the topic is one that is deeply familiar to them, they might choose to make a decision without involving other editors. Occasionally, we receive manuscripts clearly out of the journal’s scope, and between the Senior Editor and me, we may decide to decline it without external review and without involving other board members; the Senior Editor writes a decision letter that I review, approve, and send to the authors. Alternatively, the Senior Editor may decide that the paper merits review and they would be the best editor to handle it; they take on the role of Reviewing Editor and begin to solicit the peer reviewers. Most often, the Senior Editor will open a consultation session with other members of the editorial board to assess the work as a group and to reach a decision about how to proceed. This prereview consultation session is a forum to consider the novelty and impact of the work, discuss its strengths and weaknesses, raise points in favor of or against review, and identify issues that may be raised with reviewers or discussed in a decision letter. Sometimes the best expertise lies with a Reviewing or Guest Editor, and the Senior Editor may ask them to lead the prereview consultation session and ultimately decide how to proceed. Reviewing Editors and Guest Editors are distinguished essentially by workload (Guest Editors handle fewer manuscripts), but they perform equally in terms of handling manuscripts.

We also have a cohort of rotating Assistant Features Editors (AFEs); these are talented early-career scientists and, currently, all are postdocs who have undergone a rigorous selection process for their appointment to the journal (Eckardt and Meyers, 2021). While the primary role of the AFEs is to write In Brief article summaries that highlight newly published work, they may be invited to participate in prereview consultation sessions when their expertise is a strong fit for the topic of the work. The main purpose is to provide the AFEs training and mentoring in the editorial and peer-review process. AFEs do not make decisions or handle peer review—final decisions are made by regular editorial board members who consider all the input provided; however, AFEs often make valuable contributions to the discussion.

The most pleasing outcome of the prereview consultation session is that the paper is sent for external review, handled by one of the editorial board members participating in the prereview discussion who viewed the work positively. However, roughly half of the original submissions are declined without external review. The reasons for a decline without review are varied—perhaps the work is a poor fit for the journal or otherwise outside of the scope of what we publish. Insufficient novelty is a common basis for declining a manuscript without review, meaning that the impact of the discovery was judged by the editors to be weak or insufficiently interesting. We receive many manuscripts describing solid, well-executed work but for which there is little enthusiasm among multiple board members. Papers published in *The Plant Cell* must impress the readers with their novelty and be viewed as opening new lines of investigation or pushing the field forward significantly. Initiating external review of a manuscript that we judge would fare poorly is not a good use of our reviewers’ time, and typically only delays the eventual publication of a paper in another journal for which it is a better fit.

A manuscript may also be declined without review if the prereview discussion identifies fatal flaws, like poor experimental design, lack of replication, critically-important-but-missing controls, or unsatisfactory presentation of the work (poor figures, a lack of clarity in the writing, or generally poor writing). Sometimes these are correctable, and we might encourage resubmission if the authors can address the concerns. While our primary aim is to ensure that *The Plant Cell* publishes only the highest quality of work, maintaining standards set at the founding of the journal over 30 years ago, a decision to decline without review in the case of data quality concerns is also of service to the authors. There is nothing worse than having your published work called into question, particularly if the basis for this is a quality metric that can be determined from the raw, downloadable data, or from missing controls, or poor experimental design. It is to everyone’s benefit if such issues can be identified before a manuscript is reviewed.

Whatever the issue may be, we try to provide feedback on the specific points raised in our discussions so that the authors might attempt to mitigate the concerns prior to resubmitting to *The Plant Cell* or another journal—the paper is surely going to be submitted again somewhere. I believe that this is one of the attributes of *The Plant Cell* that makes it special, that even a rejection comes with insights and considered responses from experts in your field. Decision letters are written by the handling editor and read, revised, and approved first by the Senior Editor and then by me before being sent to the corresponding author. We have set our standards high, and match this with rigorous assessment by professional academics even before review. Our aim is to help the authors, only reviewing work that has a good chance to make it through to acceptance, at least from our first-pass assessment. We also want papers published in *The Plant Cell* to set the standard for the community, which is why we occasionally publish commentaries (often authored by our editorial board members) that discuss issues that we have repeatedly encountered in evaluating manuscripts. See, for example, our commentaries on BiFC (Kudla and Bock, 2016), RT-qPCR (Udvardi et al., 2018), criteria for microRNA annotation (Axtell and Meyers, 2018), or our recent commentary on epigenetic data standards (Schmitz et al., 2022).

For all manuscripts declined without review, we include by default the option for transfer to *Plant Direct*, a sound science journal jointly published by the American Society of Plant Biologists, the Society for Experimental Biology, and Wiley. The uptake for this offer is low, perhaps reflecting that if the authors had aimed for *The Plant Cell*, they may have other journals in mind as second choices before selecting a “sound science” journal. While *Plant Physiology* might be an alternative, because our criteria for consideration are not substantially different, we offer a transfer to *Plant Physiology* only for papers that are declined after external review.

Authors have the option to appeal a decline decision that did not include encouragement to resubmit. There is no stigma associated with an appeal. Editorial decisions are subjective, and we are interested to hear the authors’ perspective if they feel that we missed important points or we misclassified important work. An appeal stimulates another round of internal discussion, typically among the same participants involved in the prereview consultation, or we bring in new board members if needed. We occasionally reverse our opinions on appeal and reconsider the work, although this is the outcome in the minority of cases. The appeal letter should make a clear case against the decision to decline. My own experience is that it is best to wait at least a few days to consider your case carefully and write an appeal letter that references the major concerns from the decision letter and explains how statements therein suggest that the editors missed important points or otherwise made a mistake in their assessment.

As I read over a newly submitted, but revised, previously declined manuscript, I ask myself the following questions: Why was it resubmitted? Have the authors addressed the major concerns, performed new experiments, or pushed back against the earlier concerns? Did we encourage resubmission or not? If not, why are the authors asking us to reconsider? Was there an appeal of our earlier decision? How cogent and clear is the “Response to Reviewers” document? Was the push-back against earlier concerns appropriate? I will assign a Senior Editor to handle a prereview consultation and ask that they consider these questions. In most cases, resubmissions are handled by the same editors who assessed the original submission; however, in cases where an editor is unenthusiastic about resubmission, I will, by mutual agreement, assign it to another editor who views the work more positively.

Manuscripts for which revision was requested are sent directly to the original handling editor. We ask our editors, when possible, to judge if the authors have adequately addressed the reviewers’ concerns and to accept such manuscripts without going back to the reviewers, as this can save substantial time and effort. This is aided by a thoughtful “Response to Reviewers” document and evidence of a serious attempt to address the reviewers’ concerns. However, editors may decide that one or more of the reviewers should review the revision.

If an editor who handled your earlier work has left the board, we endeavor to keep as much continuity as possible. I’ll work with a Senior Editor to assign the manuscript to a handling editor with expertise as close as possible to the editor who originally handled it. Senior Editors typically stay on the board for a longer period of time, and thus can provide a greater measure of continuity. I also ask departing board members to stay on in a reserve capacity for 6 months, not taking new manuscripts, but remaining active in the system in case revised manuscripts are submitted that they handled previously.

A final consideration is whether we decline papers that may have been “scooped” by other groups. Some top journals decline to consider papers for which other groups have claimed precedent with an earlier, closely related publication. We recognize that a manuscript typically represents years of work, and a race to publish first at the end of an independent investment of such time by several labs likely benefits no one. However, after the passage of time, the perception of a paper similar to earlier published work shifts from “recently scooped” to “repetitive and lacking novelty”; in our case, that amount of time is roughly 6 months. We may decline papers for similarity to work published more than 6 months prior to a first submission (or a resubmission after a manuscript was declined without encouragement to resubmit), although the extent of overlap in the findings may be a matter for discussion in the authors’ cover letter. Preprints have also helped to clarify the importance of freeing researchers from the stress of being scooped, as it’s possible to show a snapshot of the status of work at a point in time, even if peer review and revision take much longer. We do not consider preprints to count as a prior publication that impacts novelty.

I hope this has given greater clarity on our processes and the consideration that is given to manuscripts that you submit to *The Plant Cell.* We are keen to publish the best work of our community, plant biologists, and through the process of evaluating your work, we try to provide helpful feedback. Ultimately, the most satisfying outcome for editors is to shepherd excellent work through the process of review and revision and to see the publication of that work and the impact of the advances that it represents. I hope your excellent work will end up in our pages.
